# Visual diagnostics for female genital schistosomiasis and the opportunity for improvement using computer vision

**DOI:** 10.1017/S0031182025100826

**Published:** 2025-12

**Authors:** Morgan E. Lemin, Amaya L. Bustinduy, Chrissy h. Roberts

**Affiliations:** Department of Clinical Research, London School of Hygiene and Tropical Medicine, London, UK

**Keywords:** artificial intelligence, computer vision, diagnostics, female genital schistosomiasis, FGS, visual

## Abstract

Female genital schistosomiasis (FGS) is a chronically disabling gynaecological condition, impacting up to 56 million women and girls, mostly in sub-Saharan Africa. In lieu of a gold standard laboratory test, it is possible to diagnose FGS visually. Visual diagnosis is performed through inspection of the cervix and surrounding tissue to identify signs of *Schistosoma* egg deposition, associated inflammation and granuloma formation. The change related to egg deposition can be very subtle and heterogeneous and is often seen in the context of other altered cervical morphology. Visual diagnostics for FGS are therefore currently highly subjective and lack specificity, with low consistency of grading between trained expert reviewers. Computer vision, driven by artificial intelligence, is an enticing prospect to overcome these issues due to the potential to accurately detect and classify the subtle changes and patterns that are indiscernible to human graders. Computer vision also offers the opportunity to support resource-constrained regions with few staff trained on visual diagnostics. However, several challenges stand in the way of progressing and successfully implementing computer vision tools for FGS. These challenges are particularly related to the variation in the appearance of the cervix (with or without disease) and FGS lesions, as well as the difficulty with accurately labelling cervical images. Exploring alternative annotation methods and model architectures is likely to improve the performance of FGS computer vision tools. This paper will explore the challenges of FGS computer vision and provide suggestions on how to overcome these barriers to enhance visual diagnostics for FGS.

## Graphical abstract

**Figure:**
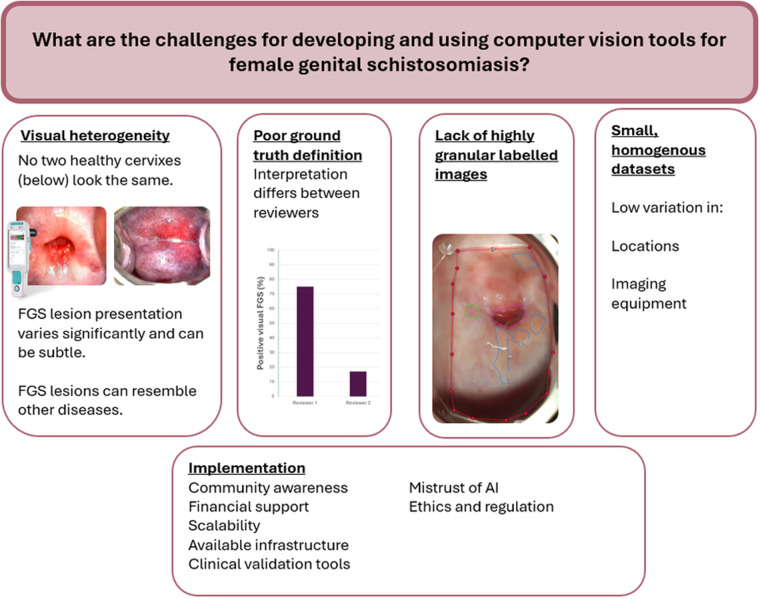

## Introduction

Female genital schistosomiasis (FGS) is a chronically disabling gynaecological condition estimated to affect up to 56 million women and girls, predominantly within sub-Saharan Africa (Bustinduy et al. 2022). FGS occurs when *Schistosoma* eggs become trapped in the tissue of the genital reproductive tract (Kjetland et al. 1996). The body’s intense inflammatory response to the presence of the eggs, and attempts to contain the infection with the formation of granulomas, is the cause of most of the associated morbidity (Kjetland et al. 2005). As part of this inflammatory response, characteristic lesions begin to form throughout the reproductive tract, including on the cervical mucosal surface and through the vaginal canal (Kjetland et al. 2014; Randrianasolo et al. 2015). The resulting symptoms have a significant overlap with the symptoms of sexually transmitted infections (STIs) and other sexual and reproductive health (SRH) conditions. These symptoms include bleeding and abnormal discharge, pain during intercourse, abdominal pain, infertility and subfertility (Kjetland et al. 2008, 2010; Hegertun et al. 2013; Bustinduy et al. 2022).

Diagnosing FGS in resource-constrained endemic settings is challenging, primarily due to the need for expensive, highly centralized equipment and extensive training (Bustinduy et al. 2022; Lamberti et al. 2024a). FGS can be diagnosed through visual examination, molecular testing for *Schistosoma* DNA or by histopathology. Nucleic acid amplification tests, such as polymerase chain reaction (PCR) on genital samples provide a sensitive and specific test for FGS; however, the test processing time and the equipment needed means PCR is not suitable as a point-of-care test (Kjetland et al. 2009; Sturt et al. 2020). Alternatively, isothermal molecular diagnostic tests such as loop-mediated isothermal application and recombinase polymerase amplification for FGS on genital samples offer a more field-friendly diagnostic test due to producing results faster with less equipment needed (Archer et al. 2020; Van Bergen et al. 2024). Histopathological examinations of cervical biopsies and circulating anodic antigen (CAA) tests are also available; however, a biopsy will only detect eggs if taken from a cervical site where the eggs have been deposited, and CAA will only indicate the presence and burden of live worms (Hoekstra et al. 2021; Nemungadi et al. 2022). Serology, to detect antibodies specific to schistosomes, and urine microscopy, to detect eggs, can be used to diagnose schistosomiasis broadly but are not definitive for FGS, as they do not necessarily confirm genital involvement (Galappaththi-Arachchige et al. 2018).

There is evidence that chronic FGS may persist after the active infection has been cleared, presenting diagnostic challenges that laboratory-based tests are not currently capable of meeting but that visual diagnostics may be well suited to overcome. While molecular and histopathological tests can be highly sensitive and specific for detecting schistosome genetic material or live worms, they may not be reliable for detecting and characterizing the chronic changes following treatment and infection clearance (Kjetland et al. 2006; Downs et al. 2011). There are indications that this chronic disease state may be more prevalent in older women (Kjetland et al. 2009; Bustinduy et al. 2022). Multiple studies have reported the pattern of younger women having higher rates of schistosome genetic material retrieval from the genital tract, while older women are more likely to present with visually detectable lesions (Kjetland et al. 2009; Sturt et al. 2020; Lamberti et al. 2024b). While there are strong indications of both an active and a chronic stage of FGS disease, no standardized definition of these stages currently exists. The presence of visually identified genital lesions in the absence of detectable active adult worm pairs or schistosome genetic material could be used to indicate a chronic stage of the disease. This chronic stage is characterized by progressive fibrosis and is the consequence of ongoing local inflammatory damage and granuloma formation around the trapped eggs. For patients in this stage, in lieu of a gold standard molecular test, visual diagnostics may remain a necessary diagnostic method (Bustinduy et al. 2022).

### The current landscape of visual diagnostics

**Figure 1.**
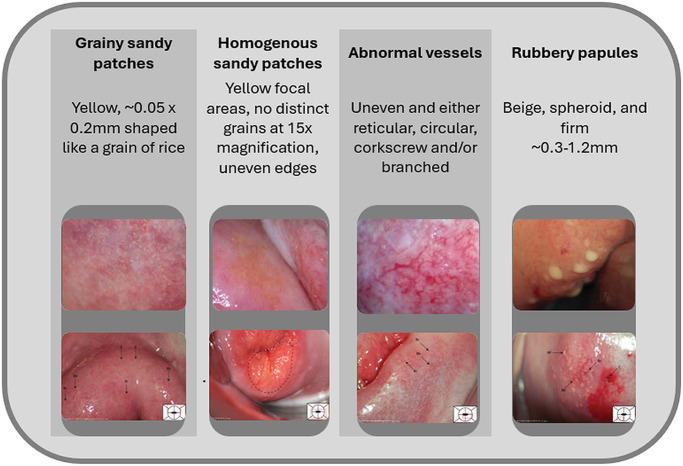
The four classic female genital schistosomiasis lesion types: Grainy sandy patches, homogenous sandy patches, abnormal vessels and rubbery papules. Images taken from the WHO FGS Pocket Atlas, 2015. The WHO FGS Pocket Atlas is licensed under CC BY-NC-SA 3.0.

The existing visual FGS diagnostic criteria, developed around 2010 and described in the **World Health Organization FGS Pocket Atlas** (2015), involve the visual identification of one or more of 4 types of lesions: grainy sandy patches, homogenous sandy patches, rubbery papules and abnormal vessels (Figure 1; Jourdan et al. 2013; Kjetland et al. 2014; Norseth et al. 2014; Randrianasolo et al. 2015). While this is currently considered sufficient visual criteria for diagnosis, the lesions are often difficult to definitively identify and may not be highly prevalent in the cervical and vaginal tissue of positive cases. The characteristic lesions of FGS on the cervix can then resemble both normal variations seen in healthy cervical tissue, and various forms of non-FGS altered cervical morphology. To further increase the difficulty in identifying these lesions, the appearance of a healthy and disease-free cervix can vary significantly depending on medical and demographic factors such as a person’s age, reproductive history and sexual history (Prendiville and Sankaranarayanan, 2017).

Visual diagnostics are typically performed with the aid of a colposcope, which is essentially a low-powered microscope with a high-powered light source (Bustinduy et al. 2022). Traditional freestanding colposcopes require stable electricity sources and are expensive pieces of equipment, costing around USD $20 000 depending on the model (Lamberti et al. 2024a). Handheld colposcopes are an alternative option. They are cheaper (around USD $4000) and are battery powered, so they can be charged to be used remotely in areas with unstable electrical infrastructure (Søfteland et al. 2021; Sturt et al. 2023; Lamberti et al. 2024a). Both types of colposcope require extensive training to operate and the price of handheld devices can still be unaffordable (Xue et al. 2020; Bustinduy et al. 2022). As a result, they are not widely available for use in FGS endemic settings, particularly outside of urban areas (Fokom Domgue et al. 2024). Other handheld devices, such as smartphones and digital cameras have been investigated for use in FGS diagnosis, but may not be useful without enhancement due to lower magnification capabilities and weaker light sources (Søfteland et al. 2021).

There are several important limitations to FGS visual diagnostics, such as the equipment costs and training needs. The fundamental limitation, however, is that visual diagnostics for FGS are highly subjective and lack specificity due to significant visual heterogeneity, as evidenced by the ‘slight’ agreement (Cohen’s kappa = 0.16) between trained expert reviewers (Sturt et al. 2023). There are also no internationally agreed clinical guidelines or standard operating procedures to guide the systematic screening, identification, grading and recording of the characteristics of FGS lesions. However, for women who continue to suffer from chronic lesions, or are in areas without access to other testing methods, visual diagnostics may represent the only opportunity for diagnosis and better management of their disease. There is, therefore, still a need for visual diagnostics for FGS and further efforts are required to reduce visual subjectivity, enhance standardization, and improve overall reliability and reproducibility.

### Why should computer vision be applied to FGS visual diagnostics?

One potential solution for the task of improving visual diagnostics is the application of computer vision, a type of artificial intelligence (AI) (Lindroth et al. 2024). Computer vision has been applied to various medical imaging modalities (chest X-ray, magnetic resonances imaging, computed tomography and ultrasound) in many clinical contexts such as dermatology, neurology, pulmonology and ophthalmology (Elyan et al. 2022). FGS computer vision models would use mathematical representations of digital images to enable computers to ‘look at’ an image and then detect or classify FGS lesions.

Computer vision encompasses supervised, unsupervised or semi-supervised learning approaches, each of which can be achieved with a wide range of model designs, known as the ‘model architecture’ (Table 1).

**Table 1.** Common methods, use cases and architecture examples for computer vision

**Computer vision**	**Key references**
**Supervised computer vision**	
The model is trained on images with associated labels, which provide information on the true state or content of an image.	(LeCun et al. 1989) (Esteva et al. 2021) (Ting et al. 2017) (He et al. 2015) (Ronneberger et al. 2015) (Dosovitskiy et al. 2020)
**Common medical use case:** Image classification, anomaly detection or segmentation when large datasets of accurately annotated images are available.	
**Common core architectures and methods:** Convolutional neural networks (CNNs) such as ResNet for classification, U-Net for image segmentation and YOLO (You Only Look Once) for object detection. Vision transformers (ViTs).	
**Performance and validation metrics:** Accuracy, precision, recall, Dice coefficient etc.	
**Example:** Guided by the image annotations, a CNN processes cervical images through layers and learns to distinguish certain visual features (e.g. edges and textures) to classify, detect or segment.	
**Unsupervised computer vision**	
The model is not provided with the associated labels and therefore learns about the images by looking for the inherent structures, patterns and features that exist.	(Goodfellow et al. 2014) (Wang et al. 2015) (Caron et al. 2020)
**Common medical use case:** Useful when annotated medical images are unavailable, annotating the images is not possible/impractical or not enough images are available to robustly train a model.	
**Common core architectures and methods:** Auto-encoders: Compress then reconstruct input images. Can use the reconstruction error to highlight areas in an image that deviate from normal patterns (i.e. anomaly detection).	
Generative adversarial network: A generator and a discriminator learn to produce new realistic images.	
**Performance and validation metrics:** Structural similarity index measure, inception score, Fréchet inception distance, reconstruction error etc.	
**Example:** A model trained on cervical images learns the distribution of normal anatomy and can then generate synthetic data or detect abnormalities (as deviations from the normal distribution).	
**Semi-supervised computer vision**	
The model is trained on a small set of labelled images, accompanied by a larger set of unlabelled images. The model is guided through the training process with the labelled data and the inherent structure of the unlabelled data.	(Ferreira et al. 2023) (Kage et al. 2024) (Wang et al. 2021) (Lee 2013)
**Common medical use case:** Used when there are sufficient unannotated images available but only a small portion of annotated images.	
**Common core architectures and methods:** Pseudo-labelling: The model generates labels that are then used for training.	
Many CNNs and ViTs can be adapted to be trained with a semi-supervised learning framework.	
**Performance and validation metrics:** Similar to supervised learning (e.g. accuracy, precision, recall and F1 score).	
**Example:** A large set of 5000 cervical images is available but only 500 images have been annotated by experts. A semi-supervised model is trained using the annotated images and the inherent structure of the unannotated images. It then generates pseudo-labels for the unannotated data.	

Supervised computer vision, currently the most common computer vision learning approach, requires that the model is trained on images that have associated labels which provide information on the true state of an image (Bishop 2006; Esteva et al. 2021; Spathis et al. 2022). The majority of supervised medical computer vision relies on convolutional neural networks (CNNs), which process the image step-by-step using multiple stages or ‘layers’ (LeCun et al. 1989; Esteva et al. 2021). A CNN typically starts with convolutional layers to scan and detect patterns, then pooling layers to reduce the image size while preserving important features, before moving on to flattening and fully connected layers to process the information and produce a final prediction. This process is designed to detect and learn patterns such as textures, edges, shapes and other visual features within the images and is used to give outputs such as disease state classification or lesion segmentation and detection (LeCun et al. 1989; Takahashi et al. 2024). There are several common CNN architectures. ResNet models are commonly used for image classification and leverage residual connections that allow the output to skip one or more layers. This means that deep networks (50+ layers) can be built without model performance decreasing as the images flow through the high number of layers (He et al. 2015), U-Net models, often used in image segmentation tasks, use an encoder-decoder framework to first ‘encode’ the image by reducing the size while keeping the important features then ‘decode’ the compressed image by gradually upscaling the important features to reconstruct the output (Ronneberger et al. 2015). Another common CNN architecture are the YOLO (You Only Look Once) models, commonly used for object detection (Wang et al. 2022). Vision transformers (ViTs) are another, comparatively newer, form of supervised computer vision, which do not scan across the images like CNNs but instead break down the images into smaller pieces to analyse the relationship between them all simultaneously (Dosovitskiy et al. 2020). Both CNNs and ViTs have their strengths and each has been found to outperform the other in various tasks; however, there does not appear to be a consistent pattern explaining why one architecture works better than the other in specific cases (Takahashi et al. 2024).

In unsupervised learning, the model is not provided with the associated labels and therefore learns about the images by looking for the inherent structures that exist within them (Bengio et al. 2013). The resultant unsupervised model would then classify images into groups that could then be labelled *post hoc* by the investigator. A generative adversarial network (GAN) is an example of a model that can be trained with an unsupervised approach (Goodfellow et al. 2014). A GAN works based on game theory by having 2 networks, a generator and a discriminator, compete with one another. The generator attempts to create images that are as photorealistic as possible and to trick the discriminator, which is attempting to spot the difference between synthetic and genuine images. Both networks become better at doing their jobs (the adversarial training process) until a selected dataset of realistic images is created (Goodfellow et al. 2014). Another example of unsupervised learning is auto-encoder anomaly detection (Hinton and Zemel, 1993; Neloy and Turgeon, 2024). In this architecture, the auto-encoder can be trained only on ‘normal’ (i.e. healthy) images to learn how to accurately recreate these images. When it is then presented with unseen images, the model will attempt to reconstruct these and measure the difference between the original image and the reconstructed image (the reconstruction error) with the theory that there will be a higher reconstruction error in the images with the anomalies compared to the ones that closely resemble the training images (Stepec and Skocaj, 2021).

Semi-supervised learning is also possible where a model is given a small set of annotated images to inform and guide the task (i.e. classification), along with a larger set of unannotated images from which it learns the inherent structure (Van Engelen and Hoos, 2020). This can be beneficial when labelled images are scarce. Many models will also use more than one architecture type in a larger pipeline. Yang et al. designed an algorithm for cervical cancer classification that was made up of a pipeline of a multi-modal GAN, U-Net and an auto-encoder (Yang et al. 2024). The choice of computer vision model architecture is usually a product of the desired output, the complexity of the task, the computational resources available and, importantly, the (annotated) data available (Elyan et al. 2022; Huang et al. 2023). Generally, multiple different architectures are tested for each task, and each model architecture comes with trade-offs. For example, the more complex models often require much higher levels of computational power or are prone to instability, while simpler models may underperform or struggle to handle complex data or tasks effectively (Van Engelen and Hoos, 2020; Esteva et al. 2021; Vargas‐Cardona et al. 2023).

The application of computer vision to the visual signs of FGS on the cervix and surrounding tissue may be a solution to many of the challenges of FGS visual diagnostics (Table 2). Several computer vision core architectures, such as ResNET50 (Liu et al. 2021) and Faster R-CNN (Hu et al. 2019), have already been trained using cervical images in the context of cervical cancer. A review of cervical cancer algorithms and their applicability to FGS was conducted in 2023 and found 13 algorithms that were ‘relevant for FGS diagnosis’; however, none of the 13 algorithms had open-source code and could not be immediately fine-tuned for FGS images (Jin et al. 2023). Cervical cancer computer vision algorithms are already in the process of being validated, after extensive research, model training and fine-tuning. The HPV-automated visual evaluation (PAVE) study is already underway to validate a screen-triage-treat approach with the support of a computer vision model (de Sanjosé et al. 2024). FGS has also been listed as a potential confounder to some cervical cancer computer vision models (Desai et al. 2022).

**Table 2.** The barriers to visual diagnostics for female genital schistosomiasis (FGS) and the potential computer vision-based solutions

Visual diagnostic barriers	Computer vision solutions
Cost and availability of colposcope equipment	Computer vision could be integrated into cheaper handheld colposcopes. Image pre-processing in algorithms (such as image upscaling) could support the use of other handheld devices such as smartphones (Ganesan et al. 2019).
Training of expert reviewers	May be able to detect markers and patterns that are indiscernible to human graders, even those with the most experience.
Highly subjective visual diagnostic criteria	Computers do not suffer from fatigue or cognitive bias and can retain objectivity over an extended repetitive task.
Lack of standardization	Alignment between centres, clinics and studies because a homogenous approach can be applied to all grading.
Poor specificity and low inter-rater agreement	Consistent results when grading the same photos multiple times. Models can be taught on huge historical datasets and can make predictions once taught. Computer vision can detect subtle variations and patterns that may not be discernible to humans.

Training a computer vision model requires significant technical expertise, along with access to high-powered computers fitted with graphics processing units that facilitate the computationally intensive process. Using the model once it is trained is simpler, often only requiring the type of central processing units found in typical personal computers and smart devices (Pietrołaj and Blok 2024). This means that it is possible to use clinical computer vision tools in resource-constrained settings, either directly on a smart device/standard computer, or via the cloud if reliable internet services are available. There is also the option to integrate computer vision tools directly within a colposcope for immediate diagnostic feedback and support for practitioners while performing live examinations.

Integrating computer vision into FGS diagnostics may have the potential to remove several of the barriers that are currently in place (Table 2). It may have the potential to alleviate some of the cost associated with colposcopy through supporting the use of other photographic equipment (e.g. smartphones) and therefore also improving access in remote areas. As a clinical support tool, computer vision could reduce the amount of high-level specialist training required. Further, computer vision may reduce the subjectivity and biases of even the most highly trained and experienced clinical grader.

### How could computer vision be used for FGS?

There are many potential use-cases for FGS computer vision. FGS models could be trained to detect lesions when presented with images from a single patient for diagnostic purposes. The computer vision could sit within a wider pipeline of diagnostic tools, with the pathway designed to be cost-effective and reflective of the natural progression of acute to chronic disease. Models could also be fed batches of images to screen and then triage patients as appropriate. There are notable parallels between cervical cancer and FGS, and cervical cancer screening programmes offer a promising point of integration for FGS computer vision tools. Another promising application of an FGS computer vision tool is that it could become an online diagnostic tool for those in previously non-endemic settings without specific FGS testing infrastructure, as migration continues and the geographical distribution of *Schistosoma haematobium* continues to expand (Lingscheid et al. 2017; Marchese et al. 2018; Salas-Coronas et al. 2020). Prognostic uses for FGS computer vision are unlikely to be useful without further research into the relationship between visual signs, symptoms and associated morbidity (Randrianasolo et al. 2015).

For now, FGS computer vision is likely to support only specific subpopulations and function at certain points of the diagnostic pathway. Computer vision tools that have been clinically validated still require the involvement of trained clinicians, rather than serving as a stand-alone replacement for clinicians or other diagnostic tools. For example, a 2021 systematic review of the use of AI for breast cancer detection found that none of the 12 studies (*n* = 131 882 women screened) provided sufficient evidence to support the use of computer vision as a stand-alone replacement for radiologists or triage systems (Freeman et al. 2021). Since then, the Mammography Screening with Artificial Intelligence randomized control trial of 105 934 women used computer vision to triage patients into a single or double reading by clinicians (Hernström et al. 2025). The results demonstrated that by using computer vision as a support tool, rather than a complete diagnostic replacement, there was an overall increase in cancer detection of 29% (95% CI: 1·09–1·15, *P*=0·0021) and a 44·2% reduction in the radiologist screening workload (Hernström et al. 2025). As another example, the PAVE study uses a risk stratification and risk-based management approach by coupling computer vision and HPV genotyping in the diagnostic pathway following a positive HPV diagnosis, rather than using solely computer vision as a stand-alone replacement (de Sanjosé et al. 2024).

**Figure 2.**
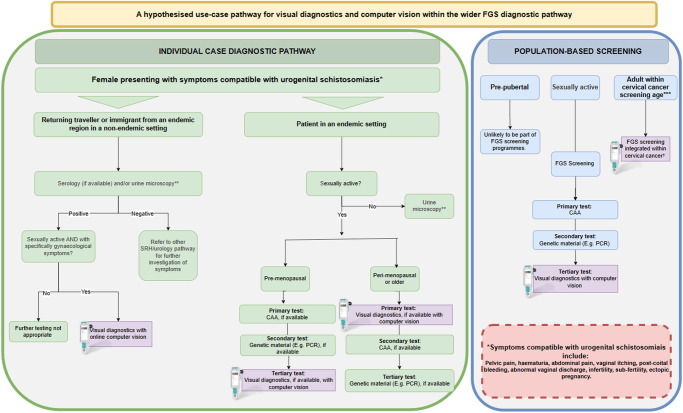
A hypothesized pathway of the potential use-cases for computer vision supported visual diagnostics within the wider FGS diagnostic pathway. Abbreviations: FGS, female genital schistosomiasis; SRH, sexual reproductive health; CAA, circulating anodic antigen, PCR, polymerase chain reaction.

A proposed diagnostic pathway is presented in Figure 2 as an example of where computer vision may fit within the wider FGS diagnostic and screening pipeline. The endemic setting pathway is based on the hypothesis that older women are more likely to test positive with visual diagnostics, based on the results of molecular versus visual diagnostics from multiple studies (Kjetland et al. 2009; Sturt et al. 2020; Lamberti et al. 2024b). As such, a sensible and cost-effective approach to the diagnostic pathway would be to start with the test most likely to be positive. Further work is needed in this area to refine the age parameters and confirm the validity of the testing pipeline.

**Urine microscopy and serology do not confirm genital involvement.***Cervical cancer screening age varies between countries.†Cervical cancer screening diagnostic algorithms vary between countries. FGS computer vision can be included in colposcopy portion of the algorithm.

## What are the main challenges to using computer vision for female genital schistosomiasis?

### Visual heterogeneity and ground truth annotation

Defining the ‘ground truth’ is one of the fundamental steps in developing a computer vision model for FGS. A ground truth is essentially the reference standard of the model and represents the ‘true state’ of what the model is trying to identify (Shen et al. 2017; Sepehri et al. 2021). In a supervised computer vision model for FGS, the ground truth determines how an image should be annotated (labelled). Without a well-defined ground truth, the line between FGS-positive and FGS-negative cases becomes blurred and incorrect classifications are built into the model (Egemen et al. 2024). An accurate ground truth is also important for the validation of unsupervised and semi-supervised models.

**Figure 3.**
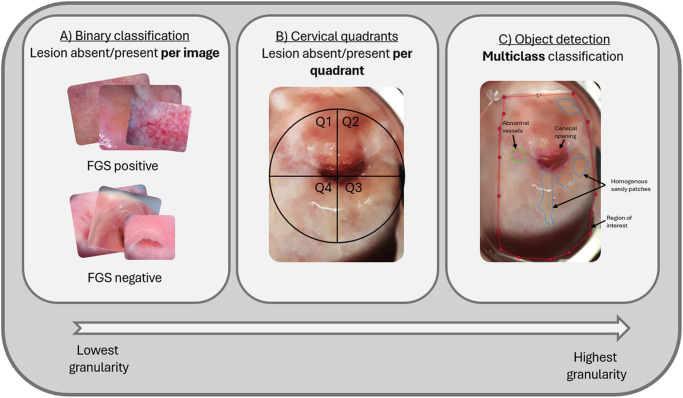
Different scales of colposcope image labelling from lowest to highest granularity. (A) binary classification (lesion present or absent) per image, (B) quadrant classification (lesion present or absent) per cervical quadrant, (C) multiclass classification, allowing for multiple features to be labelled on a single image, and for lesion size, relative location and other characteristics to be estimated. Panel C labelled using CVAT labelling software (CVAT.Ai, Palo Alto, USA).

The ground truth annotations on FGS images can be done at different scales (Figure 3). Ideally, images need to be annotated through ‘object detection’ annotation, meaning that each lesion identified is captured within a bounding box or polygon, which are then labelled (e.g. as a homogenous sandy patch; Figure 3, panel C). Higher granularity annotations, such as object detection, enable more precise model training by including only relevant lesions while excluding extraneous pixels (Ilyas et al. 2024). To the best of our knowledge, true object detection annotation has never been carried out on FGS images.

Images for FGS computer vision are currently being annotated by expert clinicians. However, this can be a laborious task, with very few people in the world both qualified and available to dedicate the time to review these images. The lack of a well-defined and easily identifiable ground truth, globally agreed grading system, associated protocols and data standards for documenting FGS lesions makes this task more daunting still. As a result, most images only have a binary classification annotation (Figure 3, panel A), with some being annotated at a cervical quadrant level (Figure 3, panel B).

Currently, the only possible guide for ground truth annotations for visual FGS diagnosis is that of the appearance of one of the four classic lesion types. However, accurately annotating if and where these lesions are present is difficult and highly subjective. For many other diseases, with similarly subjective or heterogeneous visual presentations, a suitable proxy or confirmatory molecular test is available to assist with ground truth definition. For example, computer vision-enabled screening tools for tuberculosis have been clinically validated and are already being used in places like Nigeria (Babayi et al. 2023). In this case, the model training and ground truth annotation was often being supported by bacterial culture and PCR test results (Babayi et al. 2023; Hansun et al. 2023; Scott et al. 2025). A suitable proxy or confirmatory test for FGS ground truth annotation is not always possible as laboratory tests can either be unsuitable or have low sensitivity/specificity for chronic FGS lesions that can persist after the infection has been cleared (Hoekstra et al. 2021; Nemungadi et al. 2022; Sturt et al. 2022; Lamberti et al. 2024a). If a model is only trained on images with associated confirmatory laboratory testing, then there is a risk of (A) biasing the dataset to only those who have an active infection and (B) significantly decreasing the number of images that can be fed into the model, as not all images have associated test data.

Symptom-based confirmation of FGS, where the ground truth would be confirmed with the presence of certain symptoms, is also difficult, as there is very little consistent evidence of the association between lesion and symptom presentation (Kjetland et al. 2012; Lamberti et al. 2024b). The overlap between the symptoms and endemic regions of STIs and FGS further confounds the ability contribute a symptom to either a specific FGS lesion presentation or FGS in general (Poggensee et al. 2000; Leutscher et al. 2008; Sturt et al. 2021). A Tanzanian study of 347 women found that symptom-based diagnosis of FGS had a specificity of only 15% (95% CI: 9·7–20·3%; Mbwanji et al. 2024). However, colposcopy was used as the diagnostic standard for FGS in this study, which itself lacks specificity (Sturt et al. 2023), and urine microscopy was used as a comparison and supporting diagnosis, despite the imprecise relationship between FGS and *S. haematobium* eggs in the urine (Christinet et al. 2016; Rafferty et al. 2021).

### Small and homogenous datasets

The downstream impact of issues with ground truth definition and image annotation are the comparatively small number of annotated images available to train FGS computer vision models. While some computer vision models for other diseases have been trained on hundreds of thousands or millions of images, there are currently only tens of thousands of images worldwide for FGS, and a centralized database is yet to be created. To create a computer vision model, image datasets must be broken into training, validation, and testing subsets, and blurred or obstructed images cannot be used in many training methods. This further decreases the number of ‘useful’ images that exist for the purposes of model training and testing.

The images that do exist are homogeneous because the only sources of FGS image datasets are field studies from a small number of countries, as no country currently has a screening or diagnostic programme (Ndubani et al. 2024). This can mean that the model is not sufficiently trained to handle images from other countries with different confounding diseases, sociodemographic variation or clinicians with their own imaging techniques and protocols. The lack of heterogeneity in location, photographic equipment and clinical staff can become an issue, as computer vision models can become overly specialized, performing well only on images captured in the same way and place as the images used in training (Zech et al. 2018). This means that models can exhibit a high degree of internal validity but poor external validity (Ting et al. 2017; Zech et al. 2018). A key example of this was a model being trained to detect pneumonia in chest X-rays that was also able to predict, with 100% accuracy, whether the image was taken with the inpatient portable X-ray machine or the emergency department X-ray machine (Zech et al. 2018).

## Troubleshooting the challenges in FGS computer vision

### General troubleshooting

There are many potential solutions to the challenges of poor ground truth definition, image annotation, small datasets and generalizability. Broadly speaking, adapting and utilizing different computer vision architectures may assist in overcoming the challenges in FGS computer vision. Unsupervised models are one option, which would remove the need to annotate images for the training of a model. These unsupervised models may also uncover commonalities in the images of positive cases that have not yet been uncovered by human eye detection (Patel 2019). Further, unsupervised models are potentially more generalizable as they are not trained on specific labels (Huang et al. 2023).

### Annotation troubleshooting

Label generating algorithms or ‘self-annotating’ models are an option when there are insufficient resources for manual image annotation (Huang et al. 2023). One such approach is self-supervised learning, where the model generates its own labels from the raw data it is given to then train itself in further rounds (Spathis et al. 2022). In a review of 79 studies, self-supervised computer vision models increased the overall accuracy of models by up to 29% (95% CI: 0·44%, 29·2%; Huang et al. 2023). Pseudo-labelling algorithms, a common method in semi-supervised learning, learn from the labels of a small set of annotated images and the inherent structures of a larger set of unannotated images to ‘label’ the unannotated data. Another option that shows promise for automated medical image annotation is MedSAM (Medical Segment Anything Model), a model that was trained on 1 570 263 medical images. It was created to be a pixel-level ‘universal medical image segmentation’ tool that can automatically segment medical images based on the models understanding of anatomical structures it learnt during the training phase (Ma et al. 2024). While colposcope images were not included in the original MedSAM training set, it could, in theory, be fine-tuned for the automated or semi-automated annotation of colposcope images.

### Ground truth troubleshooting

The development of effective and high-performing computer vision tools for FGS is contingent upon the development of a defined and widely accepted ground truth. Achieving this will require the collaboration of experts and the implementation of robust, standardized protocols. Expert reviewers will undoubtedly play a pivotal role in both the development and implementation of these protocols, potentially contributing without direct compensation on an often laborious task. In the absence of coordinated expert effort, model performance is likely to be compromised by inconsistent or poorly validated reference standards.

At present, an FGS visual diagnosis is binary (positive/negative) and the way that a visual diagnosis is made is not highly standardized. The World Health Organization FGS Pocket Atlas (2015) provides broad guidance on the types of lesions to identify but currently each academic team will have slightly different visual diagnostic protocols and grading scales (if one is used at all). The development of a refined visual diagnostic and grading tool, guided by the consensus of experts, might feasibly provide guidance on a more systematized classification of FGS. This grading tool could be a combination of lesion presentation (e.g. size, colour and location), patient characteristics and associated symptoms. This tool could form the basis of a more robust and standardized ground truth definition.

Redefining the annotation classes is an option when the ground truth is poorly defined. One option is to add an ‘indeterminate’ class to create a multiclass ordinal classification, rather than a binary classification of positive or negative. This introduces some flexibility within the model to handle images that are neither obvious positive nor negatives. This multiclass ordinal classification was used by Egemen et al. for a cervical cancer computer vision with positive results (Egemen et al. 2024). However, this option does risk classifying too many images as neither positive nor negative, which is unhelpful for diagnostic purposes. Soft labelling is another annotation approach, where instead of hard labelling (positive/negative) a probabilistic score is given to each label to represent the degree of uncertainty in the label. This has been used before in situations where the ground truth is ambiguous and some labels may be more likely to be correct than others (Ahfock and McLachlan 2021). By having multiple trained reviewers (3 or more) read each image, the confidence in the ground truth could be increased. If multiple reviewers used soft labelling, then a mean probabilistic score could be provided for each image.

### Small dataset troubleshooting

Using a GAN, or a similar image generation model, may be useful in improving the volume of images available for training. The GAN architecture has been applied to various medical image types such as retinal images, brain tumour MRIs and skin cancer (Ahmad et al. 2022). A GAN can also be used to deblur images, a method that was used in 2019 to deblur and enhance cervical images captured with smartphones (Ganesan et al. 2019). That paper did report an increase in detection accuracy (+21·4%); however, the model was only tested on 14 biopsy confirmed abnormal images that required manual computational blurring (as they were originally in sharp focus). Still, the paper stands as a proof of concept for the future application of this method to deblur colposcope images and therefore increase the number of useable images.

Integrating other data types (i.e. clinical, diagnostic and sociodemographic data) into the model may also improve performance and support the smaller image datasets. Liu et al. included this type of data in a model created to detect cervical pre-cancer based on colposcope images from patients in Shandong Province, China (Liu et al. 2021). This did not improve model accuracy overall but may suggest a basis for further investigation in FGS computer vision. The inclusion of this data is made possible due to the rich data sets that are being collected in field studies alongside colposcope images. By integrating and modelling other information on patients such as age, sociodemographic factors and STI status, the computer vision model may become more sensitive and specific in detecting FGS.

### Generalizability troubleshooting

The few sources of new images and proprietary ownership over these images make robust testing of the generalizability of computer vision models difficult. The generalizability of a computer vision model relates to the ability of a model to perform well on never-before-seen images, particularly images that vary from the training set based on parameters such as photographic equipment, imaging methods and geographical location. A centralized image source that showcases the photographic and geographic variation that should be included within FGS models would be highly beneficial. This would increase the generalizability of the models developed so that they could be applied to images taken from various locations and with various colposcope and imaging equipment. The benefit of doing this is highlighted by Ekem et al. in their development of a colposcope image deblurring algorithm (Ekem et al. 2025). The model was trained using images from 2 different handheld colposcope models and one freestanding colposcope from patients across 6 countries (India, Zambia, Honduras, USA, Peru and Tanzania), reflecting different sociodemographic factors and co-endemicities. The model was then tested and validated on images from Kenya and a holdout set (a portion of images set aside from the training set to later be used for testing) from all 6 countries. The model was found to be generalizable to these images (accuracy 89%), reflecting the benefit of including a variety of image sources within computer vision training sets.

Pooling images in a centralized database is likely to go a long way in improving the generalizability of these computer vision models. However, it does remain a possibility that computer vision models will need to be fine-tuned each time they are deployed in a new setting to perform at maximum capability.

## Implementation

A 2024 review of computer vision in healthcare settings found that the vast majority of computer vision models intended for clinical use are still in the development and testing phase, and that reporting and documentation on the implementation of computer vision was ‘scarce’ (Lindroth et al. 2024). Furthermore, there is currently very little information on the success of these tools on the African continent. In a review of 86 randomized control trials on the use of AI in clinical practice, only 2 were conducted in Africa (Han et al. 2024). So, while a focus on model development is important, it is equally as important to begin to take steps to ensure the successful implementation of the technology and to engage stakeholders to co-produce ideas about how these tools should be implemented safely, equitably and effectively. Taking these steps alongside model development will allow for a more expedited deployment.

The implementation of computer vision in resource-constrained settings, where FGS is endemic, poses a particular set of challenges. While there has been a huge increase in technological infrastructure (mobile phone availability, WIFI coverage and appliance charging capabilities) across the African continent, this infrastructure remains limited in many areas (Musa et al. 2023). In 2021, more than 56 000 rural hospitals in sub-Saharan Africa did not have an electrical supply (Moner-Girona et al. 2021). This, along with a paucity of financial support for the integration of these tools for neglected tropical diseases and the associated training, may make scalability difficult (The Lancet Digital 2023). Ensuring that the tools remain computationally efficient and placing them within commonly used devices (such as basic tablets or smartphones) or on the Cloud will support implementation. Keeping computer vision models open source, freely available, and compatible with multiple colposcope models and photographic devices will help keep costs down.

The ethical implications of these kinds of tools need to be considered throughout the development and implementation phases. Ethical risks include the risk to bodily autonomy and privacy, particularly through data breaches. The risk of exploitation following any data breach is also higher in this area compared to others because the highly sensitive and often stigmatizing context of SRH and FGS acts as a risk multiplier (World Health Organization 2024). The World Health Organization (WHO) has released ethical and regulatory guidance for AI and a technical brief on AI for SRH (World Health Organization, 2021, 2024). However, these broad overviews should also be accompanied by country-specific guidance that considers cultural practices, perspectives, and regulatory and legal differences (Eke et al. 2023).

Consistent informed discussion and exploration across development teams regarding the benefits and implementation of computer vision for FGS is essential. A meeting of those working on cervical cancer computer vision took place at the 38th International Papillomavirus Conference in November 2024 with the aim of standardizing approaches, developing validation criteria and identifying research gaps. A similar meeting of those working on FGS is likely to be highly beneficial.

## A pathway forward

Computer vision holds immense promise in improving the way disease is detected. While there is promise, FGS is a disease that, perhaps more than others, presents barriers to the development and implementation of computer vision. Variation in healthy cervixes and FGS lesions, along with visually confounding diseases, makes defining a ground truth difficult. This, along with very few expert image reviewers, means that at present there are few images annotated at a highly granular level. The generalizability of these models is a significant obstacle. Despite these barriers, with the AI field growing at an exponential rate, and with collaboration between teams, these tools have the potential to be successfully used in FGS.
